# Structural valve deterioration of a pericardial bioprosthesis

**DOI:** 10.1016/j.xjon.2021.10.059

**Published:** 2021-12-14

**Authors:** Hassan Kattach, Clifford W. Barlow, Sunil K. Ohri

**Affiliations:** Department of Cardiac Surgery, Southampton General Hospital, Southampton, United Kingdom

To the Editor:

**Figure:**
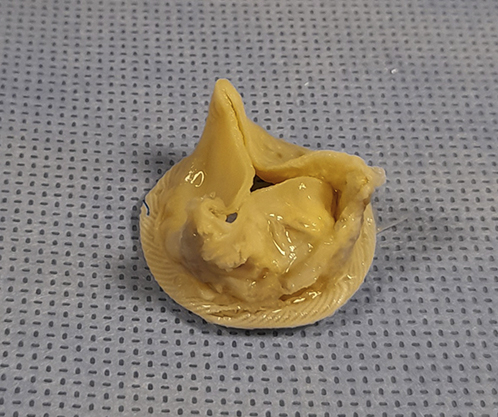

The authors reported no conflicts of interest.

The *Journal* policy requires editors and reviewers to disclose conflicts of interest and to decline handling or reviewing manuscripts for which they may have a conflict of interest. The editors and reviewers of this article have no conflicts of interest.

Lehmann and colleagues^1^ claim “excellent” outcomes for the Trifecta bioprosthesis (Abbott Structural Heart) in mid- to long-term follow-up. However, their article contains some concerns that make us question their conclusion.

First, the authors did not present any comparative evidence to support that the Trifecta bioprosthesis is noninferior to the other valves on the market. Instead, they cited published data from elsewhere, which weakens any comparison. They reported that the rate of structural valve deterioration (SVD) in their cohort is comparable with the 10% rate of SVD in the Carpentier Edwards Perimount (CEP) (Edwards Lifesciences) valve at 10 years.^2^ However, the study they cited was assessing SVD rate in a relatively young population (aged 50-65 years), whereas the authors' study population was aged 73.5 years, on average. It is well known that younger age is an important risk factor for earlier SVD. Furthermore, in a publication assessing a very large cohort of patients with a CEP^3^ (which was used as a reference in the article), the risk of SVD in patients with a CEP valve was around 0.8% at 10 years, meaning that Trifecta has 10-fold increase of the risk of SVD.

There is a growing body of evidence in the literature that suggests that Trifecta has an increased risk of early SVD^4^^,^E1, E10, E11, E12, E13 and we believe the evidence presented in their study does not support the authors' “excellent outcome” conclusion. Moreover, we question their additional conclusion that Trifecta has a “low rate of SVD” when the risk of failure at 8 years is 6.7% (1 in every 15 prostheses).

Another issue raised in the article is the success of valve-in-valve (ViV) transcatheter aortic valve implantation (TAVI) as a treatment for failed Trifecta valves. In this regard, the Trifecta is again inferior to alternative valves such as CEP because the metal stent in small sizes of Trifecta valve cannot be fractured for insertion of an adequate size of TAVI prosthesis.^5^ For this reason, ViV-TAVI is not used to treat size 19-mm failed Trifecta valves and it is seldom successful in treating size 21-mm valves. Consequently, some centers avoid implanting Trifecta prostheses smaller than 23 mm. The authors report using ViV-TAVI to treat 5 patients with size 21-mm failed Trifecta valve,^1^ but no data were presented on the residual post-TAVI transvalvular gradient, or patient–prosthesis mismatch. Our own experience in treating a patient with size 21-mm failed Trifecta valve with ViV-TAVI, and who was not fit to have a redo surgery, was disappointing because the residual gradient was 46 mm Hg.

Overall, we believe the conclusion reached by Lehmann and colleagues^1^ is not supported by their own data and it contradicts other recent peer-reviewed publications expressing concern about premature structural failure of the Trifecta valve in a significant number of patients.
